# Associations Between Audiovisual Integration and Reading Comprehension in Autistic and Non-autistic School-Aged Children

**DOI:** 10.1007/s10803-025-06960-3

**Published:** 2025-07-26

**Authors:** Grace Pulliam, Jacob I. Feldman, Mark T. Wallace, Laurie E. Cutting, Tiffany G. Woynaroski

**Affiliations:** 1 Neuroscience Graduate Program, Vanderbilt University, Nashville, TN, US; 2 Vanderbilt Brain Institute, Vanderbilt University, Nashville, TN, US; 3 Department of Hearing & Speech Sciences, Vanderbilt University, Nashville, TN, US; 4 Frist Center for Autism and Innovation, Vanderbilt University, Nashville, TN, US; 5 Vanderbilt Kennedy Center, Vanderbilt University Medical Center, Nashville, TN, US; 6 Department of Psychology, Vanderbilt University, Nashville, TN, US; 7 Department of Special Education, Vanderbilt University, Nashville, TN, US; 8 Department of Communication Sciences and Disorders, John A. Burns School of Medicine, University of Hawai’i at Mānoa, Honolulu, HI, US

**Keywords:** Autism, Reading, Audiovisual Integration, Literacy, Language, Mechanisms

## Abstract

Although not considered a core feature of autism, autistic children often present with difficulties in reading comprehension, which is a multisensory process involving translation of print to speech sounds (i.e., decoding) and interpreting words in context (i.e., language comprehension). This study tested the hypothesis that audiovisual integration may explain individual differences in reading comprehension, through its relations with decoding and language comprehension, in autistic and non-autistic children. To test our hypothesis, we conducted a concurrent correlational study involving 50 autistic and 50 non-autistic school-aged children (8–17 years of age) matched at the group level on biological sex and chronological age. Participants completed a battery of tests probing their reading comprehension, decoding, and language comprehension, as well as a psychophysical task assessing audiovisual integration as indexed by susceptibility to the McGurk illusion. A series of regression analyses was carried out to test relations of interest. Audiovisual integration was significantly associated with reading comprehension, decoding, and language comprehension, with moderate-to-large effect sizes. Mediation analyses revealed that the relation between audiovisual integration and reading comprehension was completely mediated by decoding and language comprehension, with standardized indirect effects indicating significant mediation through both pathways. These associations did not vary according to diagnostic group. This work highlights the potential role of audio-visual integration in language and literacy development and underscores the potential for multisensory-based interventions to improve reading outcomes in autistic and non-autistic children. Future research should employ longitudinal designs and more diverse samples to replicate and extend these findings.

## Introduction

Autism is a neurodevelopmental condition defined by differences in social communication and by the presence of restricted interests and repetitive behaviors, as well as sensory processing differences (American Psychiatric Association [APA], 2013). Although not considered amongst the core features of the condition, impairments in language and literacy commonly co-occur with autism (e.g., Baixauli et al., 2021; Vogindroukas et al., 2022). School-age autistic^1^ children are at risk for disproportionate difficulties with reading comprehension, which could impact their academic and future vocational success (e.g., McIntyre et al., 2017; Nation et al., 2006; Norbury & Nation, 2010). Thus, there is a pressing need to identify factors and mechanisms that may help to explain variance in reading comprehension in autism.

### Decoding and Language Comprehension Contribute To Reading Comprehension

According to the simple view of reading, reading comprehension is theorized to result from the combined contributions of decoding and language comprehension (Gough & Tunmer, 1986). A large extant literature has provided empirical support for this theory in non-autistic children (e.g., Cutting & Scarborough, 2006; Foorman et al., 2018; Hoover & Gough, 1990). While numerous factors have been hypothesized or suggested to covary with reading comprehension in autistic children, a growing body of research suggests that the simple view of reading may hold for this population, in whom decoding and language comprehension have also been found to account for the majority of the variance in reading comprehension (e.g., Brown et al., 2013; Davidson et al., 2018).

### Audiovisual Integration Likely Influences Decoding and Language Comprehension

One factor that may influence decoding and language comprehension and translate to difficulties with reading comprehension is audiovisual integration, the ability to combine information from the auditory and visual modalities in order to benefit behavior and perception. This is important to consider, given that reading is inherently a multisensory process, which requires one to rapidly and accurately convert visual information (i.e., graphemes) into auditory information (i.e., phonemes) to decode text (Squires, 2018) and to access the semantic meaning of such orthographic information to comprehend what has been read (Torppa et al., 2010). It has been theorized that audiovisual integration is foundational for a number of higher-order processes, and that altered audiovisual integration may cascade onto development across domains, including language and literacy acquisition (Cascio et al., 2016; Wallace et al., 2020).

### Audiovisual Integration Is Often Altered in Autistic Children

**Fig. 1:**
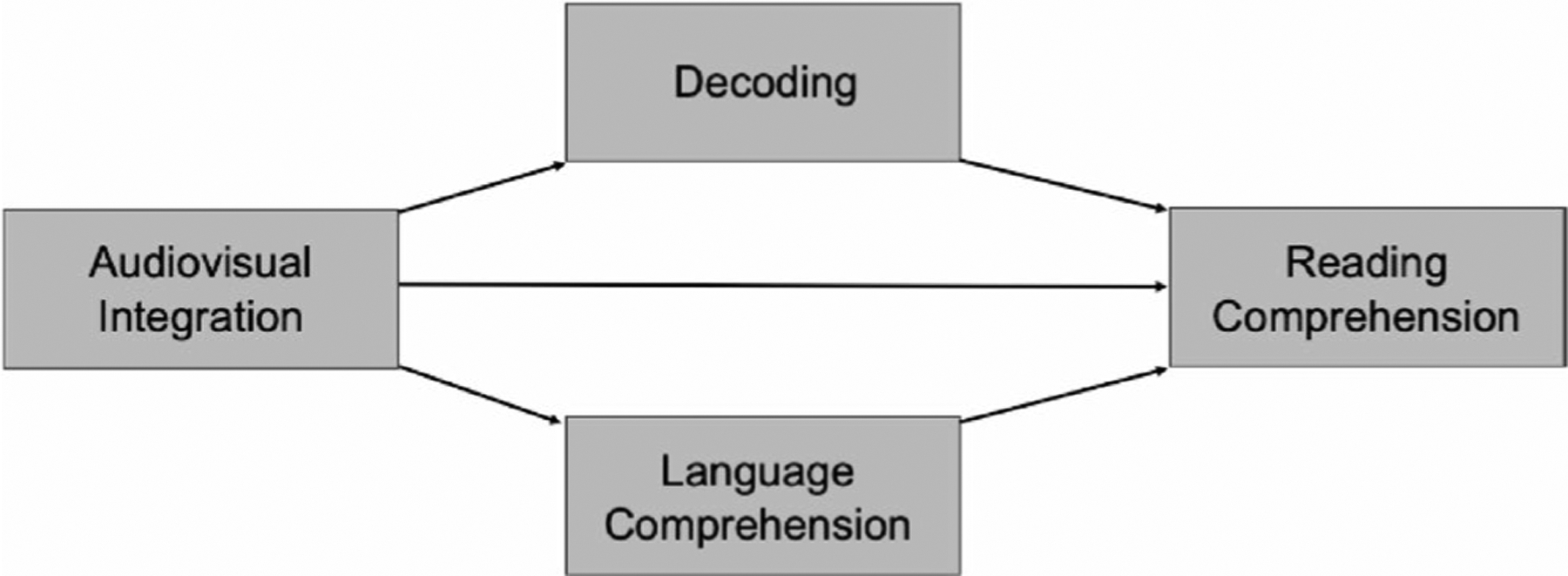
Conceptual Figure of Models Tested. *Note*. Depiction of the model tested in analyses. Audiovisual integration as indexed by the proportion of trials wherein participants reported perception of the McGurk illusion. Reading comprehension was indexed as an aggregate from the comprehension scaled score from the Gray Oral Reading Test, 5th edition (GORT; Wiederholt & Bryant, 2000) and the text comprehension scaled score of the Test of Reading Comprehension, 4th edition (TORC; Brown et al., 1978), following z-score transformation. Decoding was measured via an aggregate of (a) the fluency scaled score from the GORT, (b) the contextual fluency scaled score from the TORC, and (c) the sight word efficiency and phonemic decoding efficiency scaled scores from the Test of Word Reading Efficiency, 2nd edition (Torgesen et al., 1999) following z-score transformation. Language comprehension was indexed as an aggregate the receptive language standard score from the Clinical Evaluation of Language Fundamentals, 5th edition (Wiig et al., 2013), the standard score from the Receptive One-Word Picture Vocabulary Test, 4th edition (Martin & Brownell, 2011) and the receptive scaled score from the Vineland Adaptive Behavior Scales, 2nd edition (Sparrow et al., 2005) following z-score transformation

Children on the autism spectrum often present with differences in the processing and integration of audiovisual stimuli, in particular audiovisual speech, in comparison to non-autistic children (e.g., Stevenson et al., 2014b; Woynaroski et al., 2013; Zhou et al., 2022; see Feldman et al., 2018; Jertberg et al., 2024; Wallace et al., 2020 for reviews). Several studies have shown, for example, a reduced magnitude of audiovisual integration in response to audiovisual speech for autistic children relative to non-autistic children, when assessed by a psychophysical illusion called the McGurk effect, wherein the presentation of incongruent auditory and visual speech information (e.g., an auditory “pa” and visual “ka”) induces a fused percept (e.g., “ta” or “ha”) believed to represent integration of the cues presented across sensory modalities (e.g., Iarocci et al., 2010; Irwin et al., 2011; Mongillo et al., 2008; Stevenson et al., 2014b; see Jertberg et al., 2024 and Zhang et al., 2019 for reviews). There is mounting evidence that audiovisual integration as measured by susceptibility to the McGurk effect is linked with language comprehension in autistic children, as well as broader language and literacy skill in a range of clinical populations (e.g., Feldman et al., 2022; Pulliam et al., 2023). However, no study to date has comprehensively evaluated whether audiovisual integration is associated with reading comprehension through decoding and language comprehension in autistic and non-autistic children at school age (see Fig. 1).

### The Possibility that Diagnostic Group May Influence Relations of Interest

At the same time, it is possible that the factors contributing to reading comprehension may vary according to diagnostic group. For example, our team has previously shown that audiovisual integration as indexed by the McGurk effect may be more strongly associated with language comprehension in autistic relative to non-autistic children (Feldman et al., 2022). Other studies have shown that decoding may be less strongly associated with reading comprehension in autistic compared to non-autistic children (Henderson et al., 2014). Therefore, it is critical to consider group as a potential moderator of the relations of interest.

### Purpose

The present study, therefore, sought to evaluate whether audiovisual integration as measured by the McGurk illusion explains individual differences in reading comprehension, through its relations with decoding and/or language comprehension, in school-aged autistic and non-autistic children, with consideration of the possibility that associations of interest may vary according to diagnostic status. Our research questions were as follows:

1. Is audiovisual integration, as measured by susceptibility to the McGurk effect, associated with reading comprehension in school-aged autistic and non-autistic children? We hypothesized that increased audiovisual integration would covary with better reading comprehension.
2. Is audiovisual integration, as measured by susceptibility to the McGurk effect, also associated with decoding and language comprehension in school-aged autistic and non-autistic children? We hypothesized that increased audiovisual integration would be associated with better decoding and language comprehension.
3. Is the relation between audiovisual integration and reading comprehension mediated, or explained at least in part, by (a) decoding and/or (b) language comprehension? We hypothesized that increased audiovisual integration would be associated with better reading comprehension via increased decoding and language comprehension.
4. Are the aforementioned relations (i.e., relations between audiovisual integration and reading comprehension, decoding, and language comprehension; the full mediation model) moderated by diagnostic group? We hypothesized that some relations of interest may be moderated by group, given prior findings for differential associations between audiovisual integration, decoding, language comprehension, and/or reading comprehension for autistic versus non-autistic children.

## Methods

This study was completed at Vanderbilt University Medical Center with procedures approved by the Vanderbilt University Institutional Review Board.

### Participants

Participants were 50 autistic children (*M*_age_ = 13.1 years; 37 male, 13 female) and 50 non-autistic children (*M*_age_ = 13.0 years; 37 male, 13 female) drawn from a larger NIH-funded study of sensory functioning (e.g., Feldman et al., 2020, 2024) and matched at the group level on both chronological age and biological sex. See Table 1 for a summary of participant characteristics according to group.

Inclusion criteria for this study were: (a) chronological age between 8;0 and 17;11 years; (b) normal or corrected-to-normal vision and normal hearing, as confirmed by screening at entry to the study; (c) no history of seizure disorders; and (d) no diagnosed genetic disorders, such as Fragile X or tuberous sclerosis, per caregiver report. An additional inclusion criterion for autistic children was a diagnosis of autism spectrum disorder according to DSM-5 criteria, as confirmed by a research-reliable administration of the Autism Diagnostic Observation Schedule, 2nd edition (ADOS-2; Lord et al., 2012) and the judgement of a licensed clinician on the research team. Additional inclusion criteria for non-autistic children were: (a) scores below the screening threshold for autism concern on the Social Communication Questionnaire (SCQ; Rutter et al., 2003); (b) no immediate family members with a diagnosis of autism; (c) nonverbal cognitive ability (NVIQ), as measured by standard scores on either the Leiter International Performance Scale, 3rd edition (Leiter–3; Roid et al., 2013) or the Test of Nonverbal Intelligence, 4th edition (TONI–4; Brown et al., 2010), ≥ 85; and (d) no prior history or present indicators of psychiatric conditions or learning disorders.

### Measurement of Audiovisual Integration

Audiovisual integration was measured via a psychophysical task assessing susceptibility to the McGurk illusion (McGurk & Macdonald, 1976). This specific task was chosen because previous work from our lab showed that it yielded indices of audiovisual integration that were stable and valid for predicting core and related features of autism in school-aged autistic and non-autistic children (e.g., Dunham et al., 2020; Feldman et al., 2024). Stimulus presentation for all tasks was managed by E-Prime software.

#### Stimuli

The stimuli presented in the psychophysical task were videos of a female speaker saying the syllables “pa” and “ka” at a natural rate and volume, with neutral affect. These videos were recorded against a grey background with the speaker’s face and neck visible, in a similar manner to other stimuli used in the extant literature (e.g., Woynaroski et al., 2013). The auditory and visual tracks were separated and manipulated in Adobe Premiere to create the stimulus conditions as summarized below. Each stimulus was 1.85 s long.

#### Procedure

Participants completed the psychophysical task in a WhisperRoom (WhisperRoom Inc., Morristown, TN, USA) using a Samsung Syncmaster 2233RZ 22-inch PC monitor and Sennheiser HD550 series supra-aural headphones. Speech stimuli were presented in four conditions: auditory-only, visual-only, and congruent audiovisual speech syllables (both “pa” and “ka”), and incongruent audiovisual speech syllables (auditory “pa” and visual “ka”; i.e., McGurk stimuli). Following each stimulus presentation, participants reported what they perceived (i.e., “pa,” “ka,” “ta,” or “ha”) on a keyboard or a button box. The task consisted of 10 trials of each stimulus presented in a random order. Audiovisual integration was defined as the proportion of incongruent audiovisual speech trials in which participants reported perceiving the McGurk illusion (i.e., reported the fused percept of “ta” or “ha”). Higher values are interpreted as reflecting greater audiovisual integration and are considered more adaptive.

### Measurement of Reading Comprehension

Reading comprehension was measured via the Gray Oral Reading Test, 5th edition (GORT; Wiederholt & Bryant, 2000) and the Test of Reading Comprehension, 4th edition (TORC; Brown et al., 1978). We derived (a) the comprehension scaled score from the GORT, and (b) the text comprehension scaled score of the TORC as component variables for use in analyses.

### Measurement of Decoding

Decoding was measured via the GORT, the TORC, and the Test of Word Reading Efficiency, 2nd edition (TOWRE; Torgesen et al., 1999). We derived (a) the fluency scaled score from the GORT, (b) the contextual fluency scaled score from the TORC, and (c) the sight word efficiency and phonemic decoding efficiency scaled scores from the TOWRE as component variables for use in analyses.

### Measures of Language Comprehension

Language comprehension was measured via the Clinical Evaluation of Language Fundamentals, 5th edition (CELF; Wiig et al., 2013), the Receptive One-Word Picture Vocabulary Test, 4th edition (ROWPVT; Martin & Brownell, 2011), and the Vineland Adaptive Behavior Scales, 2nd edition (Vineland; Sparrow et al., 2005). We derived the receptive language standard score from the CELF, the standard score from the ROWPVT, and the receptive scaled score from the Vineland for use in analyses (see Table 2 for list of constructs, measures, and variables related to each research question).

### Analytic Plan

Prior to conducting analyses, all variables were evaluated for normality, specifically for skewness >|1.0| and kurtosis >|3.0|. Subsequently, missing data (ranging from 0 to 11% across variables derived for use in analyses) were imputed using the *missForest* package (Stekhoven & Bühlmann, 2012) in RStudio (R Core Team, 2023). We then evaluated whether component variables purported to tap the same construct (e.g., for decoding, language comprehension, and reading comprehension) were sufficiently intercorrelated (i.e., *r* ≥.4) to generate aggregates tapping those abilities, following z-score transformation. Scores indexing audio-visual integration were raised to the 4th power to address detected violations of normality.

A series of regression analyses was then carried out to test hypothesized associations. The PROCESS macro in R was utilized to evaluate whether the relation between audio-visual integration and reading comprehension was mediated by language comprehension and/or decoding and to test whether any associations of interest varied according to diagnostic group (in models including the putative predictor [audiovisual integration], the putative moderator [group], and the product term [audiovisual integration*group]; Hayes, 2022). Cook’s *D* was utilized to monitor for undue influence across analyses (Cook, 1977).

### Power Analyses

Power analyses were conducted with G*Power 3 and Monte Carlo power analysis simulation, given the plan for imputation of discrete missing data (Faul et al., 2009; Schoemann et al., 2017). Sensitivity analyses run in G*Power 3 specified with alpha = 0.05 and power = 0.80 revealed that we would be powered to detect unconditional and conditional relations that were small to moderate per Cohen’s (1998) criteria (i.e., *f*^*2*^ ≥ 0.11) with up to three predictors inclusive of putative predictor, putative moderator, and product terms, assuming that retention of all was warranted in final regression models, with a sample size of 100. Monte Carlo simulations specified with 1000 replications, 20,000 Monte Carlo draws per rep, and CI set to 95% indicated that we would be powered at > 0.80 (actual power ≥ 0.82) to detect parallel indirect effects assuming that paths comprising the indirect effects (*a*_*1*_*b*_*1*_ and *a*_*2*_*b*_*2*_) were at least moderate (≥ 0.3) in magnitude at *n* = 100. We were confident that we would observe effects of this magnitude based on our prior work estimating associations between audiovisual integration of speech stimuli and core and related features of autism (Feldman et al., 2018, 2022).

## Results

### Preliminary Results

Prior to running primary analyses, we ran a series of t-tests to confirm that autistic and non-autistic children displayed the expected pattern of between-group differences in response to the stimuli presented in the context of the psychophysical task. Consistent with extant studies, autistic children (*M* = 0.791, *SD* = 0.329) displayed reduced audio-visual integration in response to incongruent audiovisual speech stimuli (i.e., McGurk stimuli) in comparison to non-autistic children (*M* = 0.941, *SD* = 0.121), *t*(77.212) = 5.830, *p* <.001. This effect was moderate in magnitude (Cohen’s *d* = 0.60). Autistic children (*M* = 0.494, *SD* = 0.173) also displayed reduced accuracy in response to auditory-only stimuli relative to non-autistic children (*M* = 0.614, *SD* = 0.148), *t*(95.167) = −3.985, *p* <.001. This effect was moderate in magnitude (Cohen’s *d* = 0.75). Groups did not differ in their accuracy in response to visual-only stimuli (autistic children: *M* = 0.787, *SD* = 0.124; non-autistic children: *M* = 0.771, *SD* = 0.134; *t*(97.186) = 0.857, *p* =.394) or congruent audiovisual speech stimuli (autistic children: *M* = 0.951, *SD* = 0.058; non-autistic children: *M* = 0.970, *SD* = 0.053; *t*(97.014) = −1.579, *p* =.118). These effects were negligible to small in magnitude (Cohen’s *d* = 0.12 and 0.32, respectively).

### Generation of Aggregate Scores

All component variables used to tap reading comprehension, decoding, and language comprehension were sufficiently intercorrelated to support aggregation (i.e., with all *r* values ≥ 0.5, *p* <.001; see Table 3).

### RQ1: The Association Between Audiovisual Integration and Reading Comprehension

**Fig. 2:**
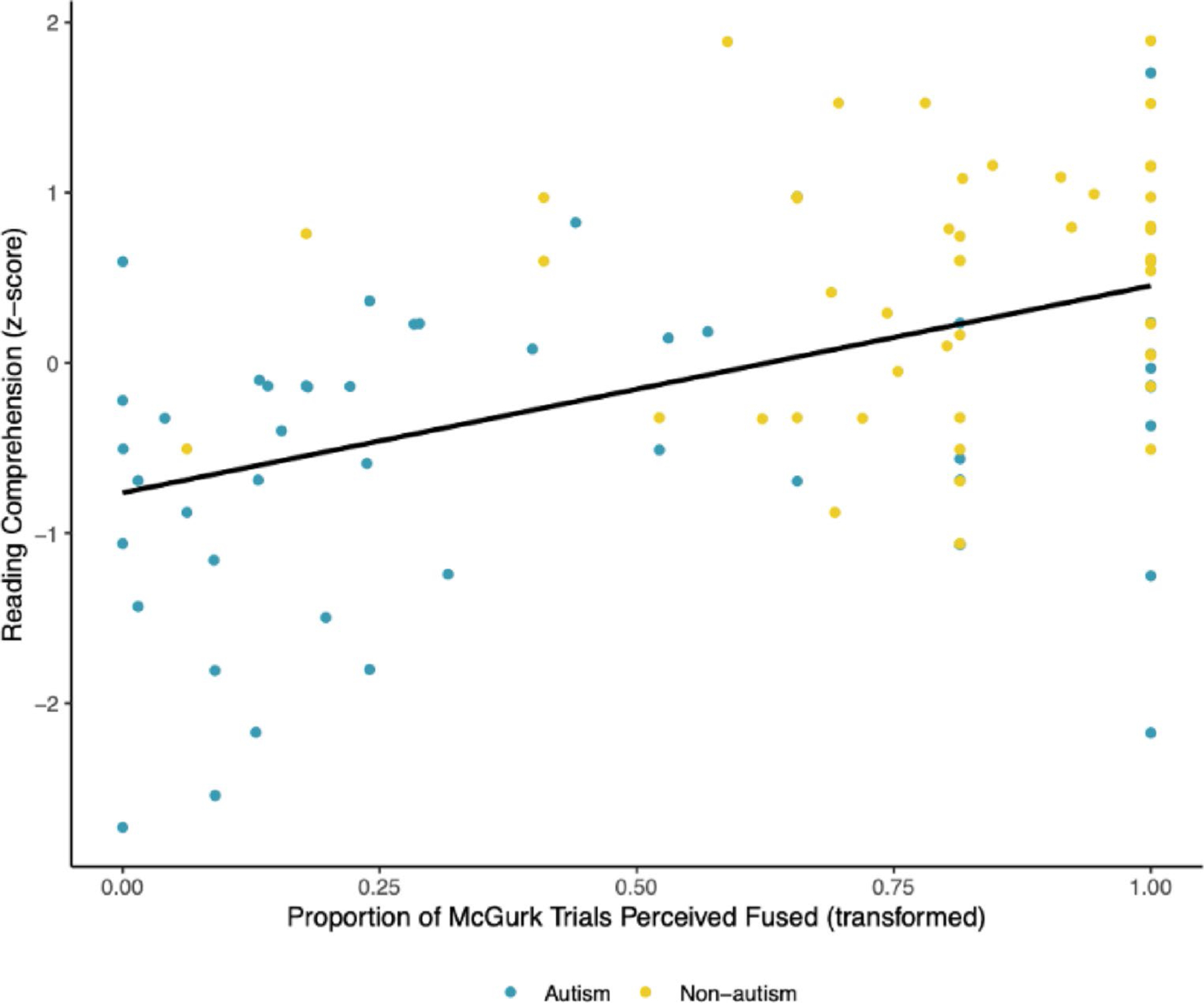
Scatterplot Depicting the Relation Between Audio-visual Integration and Reading Comprehension. Audiovisual integration as indexed by the proportion of trials wherein participants reported perception of the McGurk illusion was significantly associated with reading comprehension, with a moderate effect size. Audiovisual integration was transformed using a quartic transformation. This association did not vary according to diagnostic group

The association between audiovisual integration and reading comprehension was statistically significant (*β* = 0.458, *p* <.001; see Fig. 2). Specifically, increased audiovisual integration (i.e., increased reported perception of the fused percept of “ta” or “ha” in response to incongruent audiovisual speech trials) covaried with better reading comprehension abilities across groups. This association was moderate in magnitude.

### RQ2: Associations for Audiovisual Integration with Decoding and Language Comprehension

**Fig. 3:**
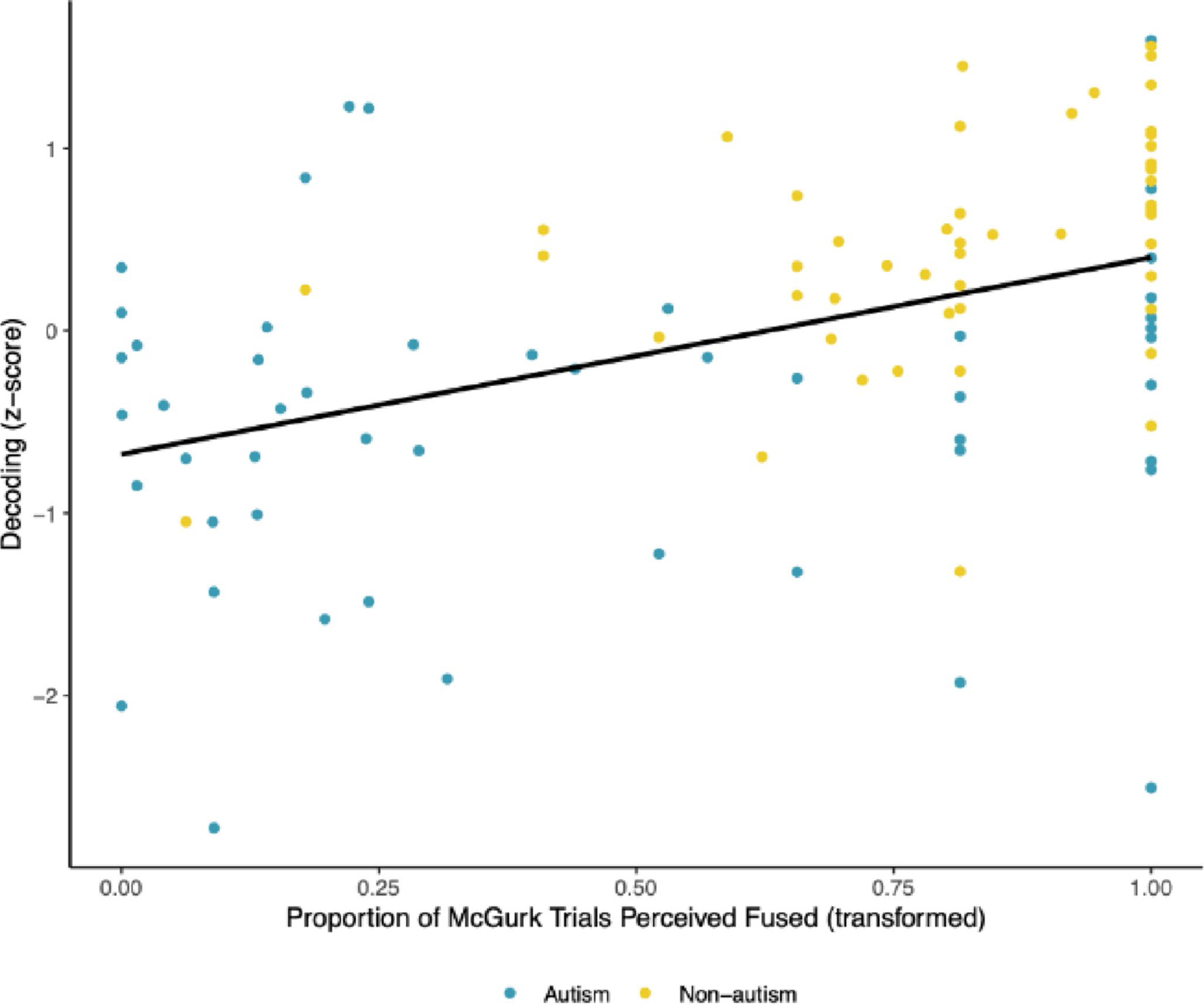
Scatterplot Depicting the Association Between Audiovisual Integration and Decoding. Audiovisual integration as indexed by the proportion of trials wherein participants reported perception of the McGurk illusion was significantly associated with decoding, with a moderate effect size. Audiovisual integration was transformed using a quartic transformation. This association did not vary according to diagnostic group

**Fig. 4:**
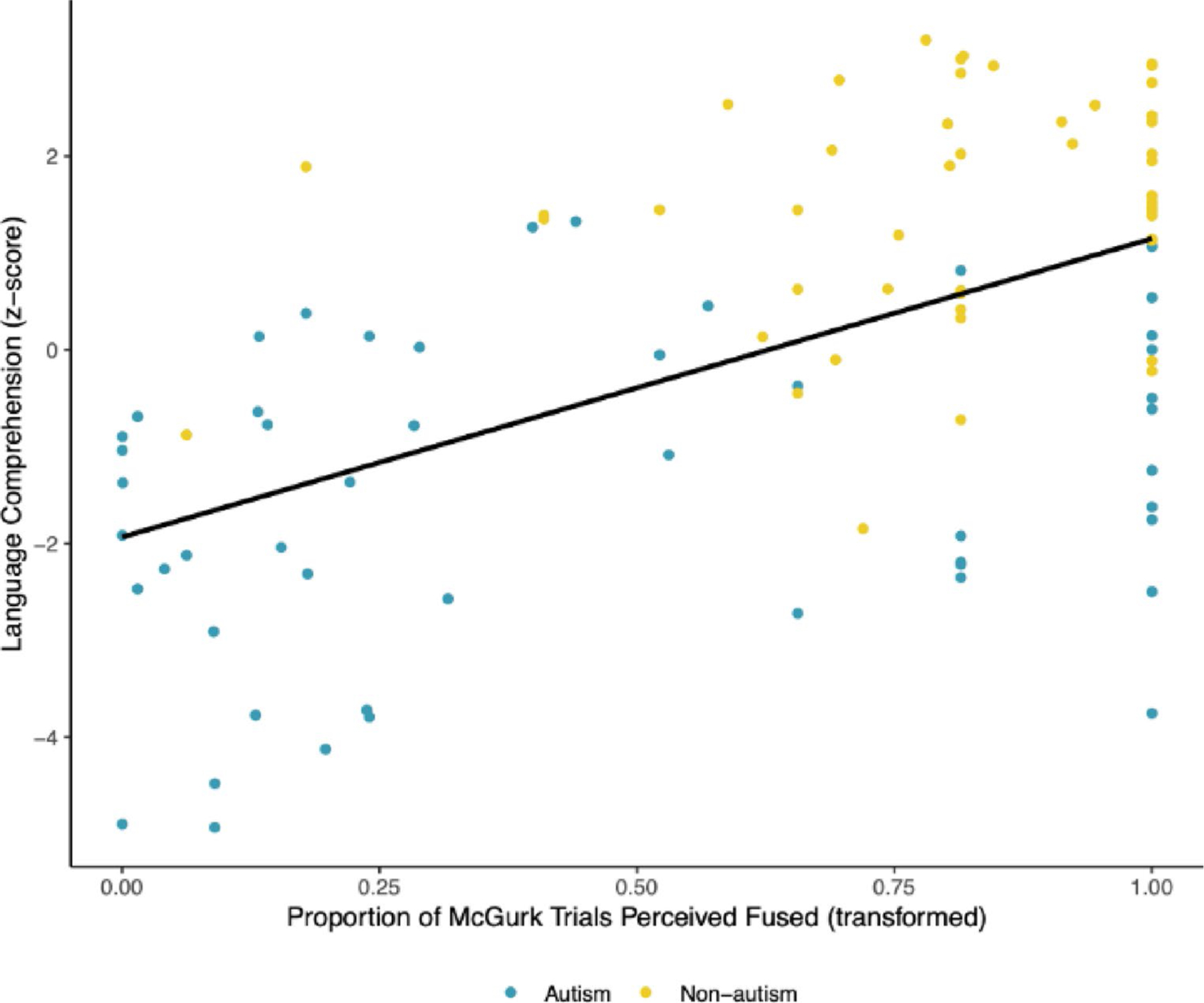
Scatterplot Depicting the Association Between Audiovisual Integration and Language Comprehension. Audiovisual integration as indexed by the proportion of trials wherein participants reported perception of the McGurk illusion was significantly associated with language comprehension, with a large effect size. Audiovisual integration was transformed using a quartic transformation. This association did not vary according to diagnostic group

Audiovisual integration was also significantly associated with decoding (*β* = 0.431, *p* <.001; see Fig. 3) and with language comprehension (*β* = 0.536, *p* <.001; see Fig. 4), such that increased audiovisual integration covaried with better decoding and language comprehension across groups. These associations were moderate and large in magnitude, respectively.

### RQ3: Tests of Mediation

**Fig. 5:**
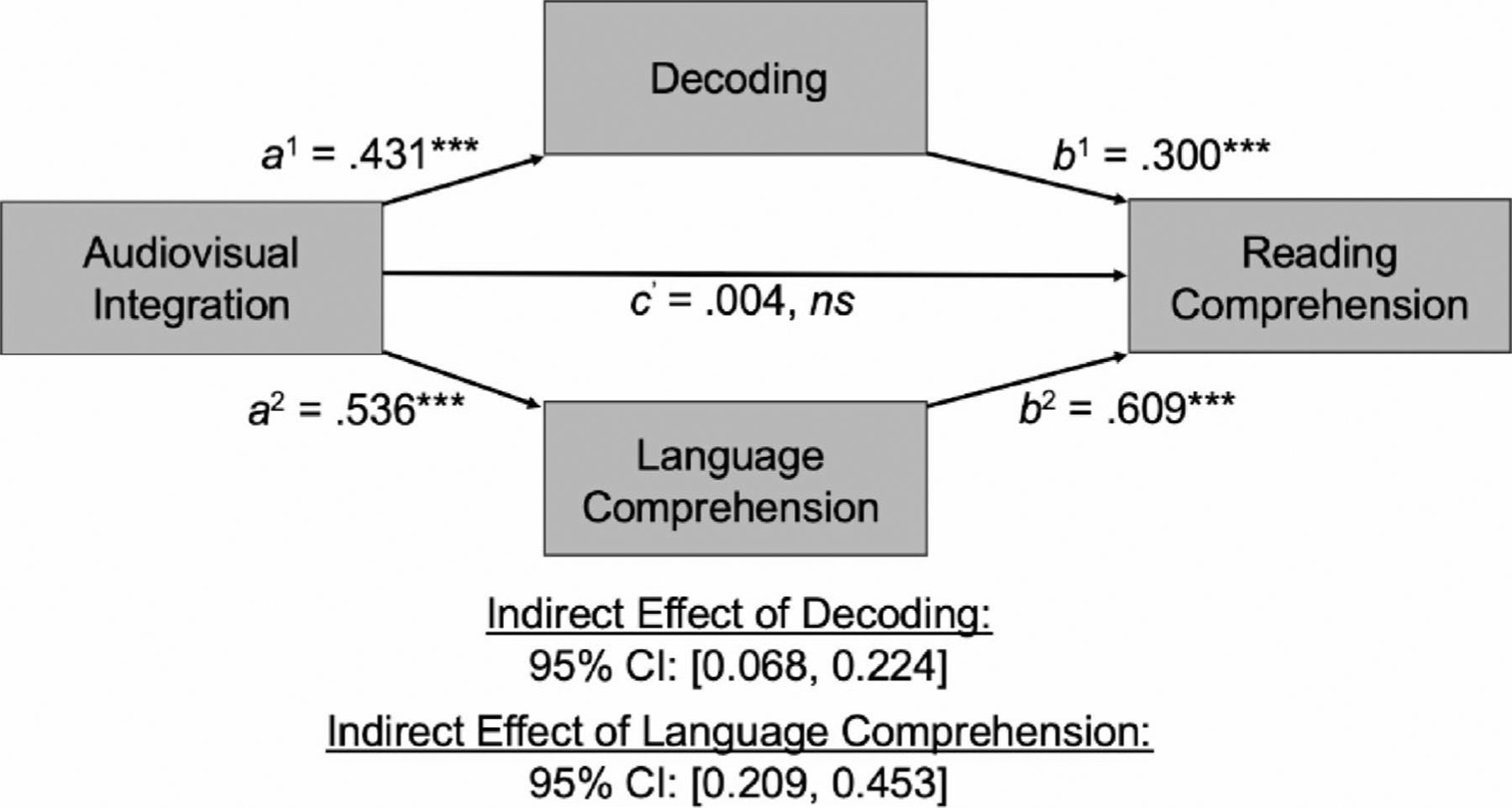
Depiction of Parallel Mediation Model. The indirect effect of audiovisual integration on reading comprehension via decoding and language comprehension. All values are standardized coefficients. *a*^1^ = the relation between audiovisual integration and decoding, not controlling for any other factors; *b*^1^ = the relation between decoding and reading comprehension, controlling for audiovisual integration and language comprehension; *a*^2^ = the relation between audiovisual integration and language comprehension, not controlling for any other factors; *b*^2^ = the relation between language comprehension and reading comprehension, controlling for audiovisual integration and decoding; *c’* = the direct relation between audiovisual integration and reading comprehension, controlling for decoding and language comprehension. Both indirect effects comprising this model, as well as the parallel indirect effect, were statistically significant, meaning that the total relation between audiovisual integration and reading comprehension was significantly reduced when controlling for the putative mediator/s. In this case, the direct relation was statistically non-significant when controlling for the mediators of interest, meaning that the relation between audiovisual integration and reading comprehension is completely mediated, or explained, by decoding and language comprehension. ***p <.001, *ns* nonsignificant

We subsequently tested whether decoding and language comprehension mediated the relation between audiovisual integration and reading comprehension via a parallel mediation model, which comprises the indirect relation between audiovisual integration and reading comprehension through decoding, the indirect relation between audiovisual integration and reading comprehension through language comprehension, and the indirect relation between audiovisual integration and reading comprehension though both decoding and language comprehension in parallel. The results of this parallel mediation model are summarized in Table 4 and depicted in Fig. 5.

The indirect relation between audiovisual integration and reading comprehension through decoding includes (a) the relation between audiovisual integration and decoding, not controlling for any other factors (the *a*^1^ path in this model); and (b) the relation between decoding and reading comprehension, controlling for audiovisual integration and language comprehension (the *b*^1^ path in this model). As reported above, audiovisual integration was significantly associated with decoding (*ß* = 0.431, *p* <.001). Additionally, decoding was significantly associated with reading comprehension, controlling for audiovisual integration and language comprehension (*ß* = 0.300, *p* <.001). The completely standardized indirect relation of audiovisual integration on reading comprehension through decoding was statistically significant, 95% CI: [0.068, 0.224].

The indirect relation between audiovisual integration and reading comprehension through language comprehension includes (a) the relation between audiovisual integration and language comprehension, not controlling for any other factors (the *a*^2^ path in this model); and (b) the relation between language comprehension and reading comprehension, controlling for audiovisual integration and decoding (the *b*^2^ path in this model). As reported above, audiovisual integration was significantly associated with language comprehension (*ß* = 0.536, *p* <.001). Additionally, language comprehension was significantly associated with reading comprehension, controlling for audiovisual integration and decoding (*ß* = 0.609, *p* <.001). The completely standardized indirect effect of audiovisual integration on reading comprehension through language comprehension was statistically significant, 95% CI: [0.209, 0.453].

The indirect effect of audiovisual integration on reading comprehension via decoding and language comprehension in parallel was also statistically significant, 95% CI: [0.314, 0.588]. The direct effect of audiovisual integration on reading comprehension, controlling for decoding and language comprehension, was non-significant (*ß* = 0.004, *p* =.953), meaning that the association between audiovisual integration and reading comprehension was completely mediated, or explained, by decoding and language comprehension across groups.

### RQ4: Planned Tests of Moderation According to Diagnostic Group

None of the relations or indirect effects of interest significantly varied according to diagnostic group. The *p* values for product terms were all > 0.05 in regression models testing moderated associations, and confidence intervals testing moderated mediation relations all included 0.

### Post-Hoc Analyses

As our sample of autistic and non-autistic children was heterogeneous, we ran a series of post-hoc analyses to explore whether the associations and indirect effects of interest here were robust to controlling for chronological age, NVIQ, and diagnostic group. All of the associations of interest and indirect effects remained significant when controlling for these variables. The results of these models are summarized in Tables S1, S2, and S3 (see Supplemental Materials).

We also considered whether the relations of interest here were specific to audiovisual integration or perhaps may hold for accuracy in perceiving auditory-only, visual-only, and congruent audiovisual stimuli that were also presented in the context of our psychophysical task. Associations between perceptual accuracy in other stimulus conditions and criterion variables of interest to this study (i.e., decoding, language comprehension, and reading comprehension) were all negligible to small in magnitude. The results of all zero-order associations across and within groups are summarized in Table S4 to facilitate meta-analyses and planning for future primary studies that may be conducted with autistic and/or non-autistic children.

## Discussion

The present study investigated the degree to which and the mechanisms by which audiovisual integration is related to reading comprehension in autistic and non-autistic children. Our results indicate that increased audiovisual integration is associated with better reading comprehension through its relations with greater decoding abilities and language comprehension. These associations did not vary according to diagnostic group but rather were observed across autistic and non-autistic children.

### Implications for Research, Theory, and Practice

Findings from this investigation extend the large and growing body of literature highlighting potential links between audiovisual integration, language, and literacy skills, and their neural substrates, in autistic children and other clinical and at-risk populations, including children with or at elevated likelihood for developmental language disorder and dyslexia (e.g., Bastien-Toniazzo et al., 2010; Calabrich et al., 2021; Edwards et al., 2018; Frei et al., 2025; Harrar et al., 2014; Meronen et al., 2013; Pulliam et al., 2023; Wang et al., 2020). The current results also lend increased empirical support for the simple view of reading (Hoover & Gough, 1990), as well as the theory that altered audiovisual integration can cascade onto the development of higher-order skills in both autistic and non-autistic children (Wallace et al., 2020). This work additionally has implications for clinical practice, suggesting that measuring multisensory integration may prove useful for predicting future difficulties in language and literacy acquisition and that multisensory approaches to intervention may prove efficacious for facilitating optimal language and literacy outcomes (e.g., Kujala et al., 2001; Magnan, 2006; Veuillet et al., 2007), though these hypotheses must be rigorously tested (Hazaymeh & Khasawneh, 2025; Schlesinger & Gray, 2017).

### Summary of Strengths

This work is the first to test associations between audiovisual integration and reading comprehension, through decoding and language comprehension, and to consider whether these associations differ based on diagnostic group in autistic and non-autistic children. There are several notable strengths of our study. First, we recruited a sample of autistic and non-autistic children that was well-matched on several characteristics, including biological sex and chronological age. This sample was adequate in size for us to test relations of interest using advanced analytic approaches and complex (mediation and moderation) models, unveiling the mechanisms by which audiovisual integration is linked with reading comprehension across our populations of interest. We additionally employed multiple measures to tap constructs of interest to the extent possible (i.e., for reading comprehension, decoding, and language comprehension), which increased the stability, and thus the predictive validity, of the aggregate scores utilized in analyses, and limited the number of statistical tests to be run and, thereby, the family-wise error of the study (Rushton et al., 1983).

### Limitations and Future Directions

There are, however, several limitations to our study. First, our use of a concurrent correlational design limits our ability to draw firm conclusions regarding the direction or causal nature of the relations we observed. Future work using longitudinal correlational and/or experimental designs is much needed to increase our confidence in the directionality or causality of links between audiovisual integration and later language comprehension, decoding, and reading comprehension.

We also only leveraged one measure of audiovisual integration, the McGurk effect. Future research could consider more measures of audiovisual processing and integration (e.g., other psychophysical or neural measures) as putative predictors of language and literacy skill (Stevenson et al., 2014a). Employing such paradigms in future work may help us to determine whether these associations are specific to the McGurk task, or if they may generalize to other aspects of processing, binding, and integration of syllables or speech as presented in a more natural and continuous manner (e.g., Van Engen et al., 2022). Further, the specific McGurk task that we used involved a closed response set, wherein participants were limited to reporting percepts presented on the button box. Going forward, researchers should consider alternative response options that permit reporting a range of potential percepts while being mindful to still limit challenges in reporting that could arise due to speech, language, imitation, and/or fine motor impairments in autistic children (i.e., asking autistic children to repeat or type more extensively on a keyboard to report their perception).

Given known differences in visual attention in autism and the high rates of co-occurring attention-deficit/hyper-activity disorder (ADHD) in autistic children, it is also important to consider broader attentional factors that could have contributed to individual differences in performance on the McGurk task that we observed (e.g., Ioannou et al., 2020; Seernani et al., 2021). In the present study, the presence of comorbid ADHD was not evaluated, and eye gaze was not monitored beyond having a member of the research team watch to ensure that participants were looking towards the computer monitor throughout the psychophysical task. Investigators should plan a priori for the assessment of ADHD symptomatology, ideally in a dimensional manner, and work to incorporate eye tracking technology into their tasks tapping audiovisual integration.

Finally, these findings may not generalize to all children on the entire autism spectrum, as the participants in our study were predominantly White and non-Hispanic and all participants were able to complete psychophysical testing and were relatively cognitively and linguistically able. Subsequent studies should increase purposive recruitment efforts and employ measurement approaches that are lower in demand (e.g., passive biobehavioral measures of audio-visual integration) to boost sample diversity and ensure the generalizability of these findings to the broader population of autistic and non-autistic children.

## Conclusion

We found a significant relation between audiovisual integration and reading comprehension that was fully explained by decoding and language comprehension across autistic and non-autistic children. These results advance our understanding of the intricate relations between audiovisual integration, language, and reading, underscoring the need to consider multisensory processes in language and literacy development and to conduct rigorous research evaluating multisensory approaches as a potential means to promote more optimal language and literacy outcomes for children who are and are not on the autism spectrum.

## Supplementary Material

supplementary material

**Supplementary Information** The online version contains supplementary material available at https://doi.org/10.1007/s10803-025-06960-3.

## Figures and Tables

**Table 1:** Summary of participant characteristics at study entry according to group

	Autism (*n* = 50) M (SD)	Non-autism (*n* = 50) M (SD)
Chronological Age (Years)	13.1 (3.1)	13.0 (2.7)
Nonverbal IQ*	109.0 (16.2)	118.0 (13.0)
Biological Sex	*n* (%)	*n* (%)
Male	37 (74%)	37 (74%)
Female	13 (26%)	13 (26%)
Race	*n* (%)	*n* (%)
Asian	3 (6%)	1 (2%)
Black/African American	2 (4%)	4 (8%)
White	35 (70%)	40 (80%)
Multiple Races	7 (14%)	5 (10%)
Not Reported	3 (6%)	0 (0%)
Ethnicity	*n* (%)	*n* (%)
Hispanic or Latino	5 (10%)	5 (10%)
Not Hispanic or Latino	43 (86%)	44 (88%)
Not Reported	2 (4%)	1 (2%)

Nonverbal IQ = Nonverbal intelligence as measured by the Leiter International Performance Scale, 3rd edition or the Test of Nonverbal Intelligence, 4th edition (Brown et al., 2010; Roid et al., 2013)

* Denotes groups significantly differed, *p* <.01

**Table 2:** Summary of constructs and variables used in analyses

Construct	Variable Used in Analyses	Role
Reading Comprehension	Average of z-scores for:	Dependent Variable (RQ1,3,4)
	(a) GORT comprehension scaled score	
	(b) TORC text comprehension scaled score	
Decoding	Average of z-scores for:	Dependent Variable (RQ2); Mediator (RQ3&4)
	(a) GORT fluency scaled score	
	(b) TORC contextual fluency scaled score	
	(c) TOWRE sight word efficiency scaled score	
	(d) TOWRE phonemic decoding efficiency scaled score	
Language Comprehension	Average of z-scores for:	Dependent Variable (RQ2); Mediator (RQ3&4)
	(a) CELF receptive language standard score	
	(b) ROWPVT standard score	
	(c) Vineland receptive scaled score	
Audiovisual Integration	Proportion of incongruent audiovisual speech trials in which the participant perceived the McGurk Illusion and reported a fused percept	Independent Variable (RQ1,2,3,4)
Diagnostic Group	Presence/absence of autism, dichotomized according to ADOS-2 and clinical interview	Putative Moderator (RQ4)

*RQ* research question, *GORT* Gray Oral Reading Test, 5th edition (Wiederholt & Bryant, 2000), *TORC* Test of Reading Comprehension, 4th edition (Brown et al., 1978), *TOWRE* Test of Word Reading Efficiency, 2nd edition (Torgesen et al., 1999), *CELF* Clinical Evaluation of Language Fundamentals, 5th edition (Wiig et al., 2013), *ROWPVT* Receptive One-Word Picture Vocabulary Test, 4th edition (Martin & Brownell, 2011), *Vineland* Vineland Adaptive Behavior Scales, 2nd edition (Sparrow et al., 2005), *ADOS-2* Autism Diagnostic Observation Schedule, 2nd edition (Lord et al., 2012)

**Table 3:** Intercorrelations of component variables used to generate aggregate scores

Component Variable	1	2	3	4	5	6	7	8	9
Variables Purported to Tap Reading Comprehension									
1. GORT Comprehension	–								
2. TORC Text Comprehension	0.79***	–							
Variables Purported to Tap Decoding									
3. GORT Fluency	–	–	–						
4. TORC Contextual Fluency	–	–	0.69***	–					
5. TOWRE Sight Word Efficiency	–	–	0.77***	0.70***	–				
6. TOWRE Phonemic Decoding Efficiency	–	–	0.86***	0.66***	0.74***	–			
Variables Purported to Tap Language Comprehensioi									
7. CELF Receptive Language	–	–	–	–	–	–			
8. ROWPVT	–	–	–	–	–	0.83***		–	
Standard									
9. Vineland Receptive	–	–	–	–	–	0.57***		0.50***	–

*GORT* Gray Oral Reading Test, 5th edition (Wiederholt & Bryant, 2000); *TORC* Test of Reading Comprehension, 4th edition (Brown et al., 1978); *TOWRE* Test of Word Reading Efficiency, 2nd edition (Torgesen et al., 1999), *CELF* Clinical Evaluation of Language Fundamentals, 5th edition (Wiig et al., 2013), *ROWPVT* Receptive One-Word Picture Vocabulary Test, 4th edition (Martin & Brownell, 2011), *Vineland* Vineland Adaptive Behavior Scales, 2nd edition (Sparrow et al., 2005)

*** *p*<.001

**Table 4:** Summary of significant parallel mediation model

Model/Variable	B (SE)	*β*	t	*p*	*f* ^2^
Model 1: Decoding (*a*^1^ path)					
1. Constant	− 0.677(0.165)	–	− 4.11	< 0.001***	–
2. Audiovisual Integration	1.080(0.229)	0.431	4.72	< 0.001***	0.237
Model 2: Language Comprehension (*a*^2^ path)					
1. Constant	− 1.933(0.354)	–	− 5.46	< 0.001***	–
2. Audiovisual Integration	3.082(0.491)	0.536	1.28	< 0.001***	0.403
Model 3: Reading Comprehension (*b* and *c’* paths)					
1. Constant	− 0.006(0.112)	–	− 0.054	0.958	–
2. Audiovisual Integration	0.010(0.162)	0.004	0.059	0.953	< 0.001
3. Decoding	0.317(0.086)	0.300	3.706	< 0.001***	0.099
4. Language Comprehension	0.280(0.040)	0.609	7.040	< 0.001***	0.593

Coefficients, *p* values, and *f*^*2*^ values for regression analyses. *f*^2^ ≥ 0.02 indicates a small effect size, *f*^2^ ≥ 0.15 indicates a moderate effect size, *f*^2^ ≥ 0.35 indicates a large effect size (Cohen, 1988)

*** *p* value for effect < 0.001
