# Supplementary material for: Associations Between Audiovisual Integration and Reading Comprehension in Autistic and Non-autistic School-Aged Children
Source: J Autism Dev Disord. Author manuscript; Available in PMC 2026 Apr 9. (PMC13063400; doi:10.1007/s10803-025-06960-3)
Supplement: supplementary material [file NIHMS2162432-supplement-supplementary_material.docx]

**Table S1**

*Summary of Parallel Mediation Model with Group Included as a Covariate*

| Model/Variable | *B* (SE) | *β* | *t* | *p* | *f* ^2^ |
| --- | --- | --- | --- | --- | --- |
| Model 1: Decoding (*a*^1^ path) | | |  |  |  |
| 1. Constant | 0.004 (.226) | — | 0.169 | .986 | — |
| 1. Audiovisual Integration | 0.567 (.246) | .226 | 2.306 | .023* | 0.054 |
| 1. Group | -0.719(.175) | -.403 | -4.104 | <.001*** | 0.194 |
|  |  |  |  |  |  |
| Model 2: Language Comprehension (*a*^2^ path) | | |  |  |  |
| 1. Constant | 0.444 (.410) | — | 1.083 | .282 | — |
| 1. Audiovisual Integration | 1.293 (.447) | .273 | 2.892 | .005** | 0.081 |
| 1. Group | -2.511 (.319) | -.367 | -7.880 | <.001*** | 0.156 |
|  |  |  |  |  |  |
| Model 3: Reading Comprehension (*b* and *c’* paths) | | | | | |
| 1. Constant | 0.264 (.141) | — | -1.863 | .066 | — |
| 1. Audiovisual Integration | 0.184(.159) | .039 | 0.652 | .517 | 0.002 |
| 1. Decoding | 0.292 (.083) | .276 | 3.514 | <.001*** | 0.082 |
| 1. Language Comprehension | 0.350 (.046) | .761 | 7.667 | <.001*** | 1.376 |
| 1. Group | 0.397 (.140) | .210 | 2.834 | .006** | 0.046 |
|  |  |  |  |  |  |
| Completely standardized indirect effect through decoding 95% CI: [0.001, 0.139] | | | | | |
| Completely standardized indirect effect through language comprehension 95% CI: [0.056, 0.290] | | | | | |
| Completely standardized parallel indirect effect 95% CI: [0.065, 0.380] | | | | | |

*Note*. Coefficients, *p* values, and *f ^2^* values for regression analyses. *f* ^2^ ≥ .02 indicates a small effect size, *f* ^2^ ≥ .15 indicates a moderate effect size, *f* ^2^ ≥ .35 indicates a large effect size (Cohen, 1988).

**p* value for effect < .05, ***p* value for effect < .01, ****p* value for effect < .001.

**Table S2**

*Summary of Parallel Mediation Model with Age Included as a Covariate*

| Model/Variable | *B* (SE) | *β* | *t* | *p* | *f* ^2^ |
| --- | --- | --- | --- | --- | --- |
| Model 1: Decoding (*a*^1^ path) | | |  |  |  |
| 1. Constant | -0.686 (0.389) | — | -1.765 | <.001*** | — |
| 2. Audiovisual Integration | 1.079 (0.234) | .431 | 4.604 | <.001*** | 0.481 |
| 3. Age | 0.000 (0.002) | .002 | 0.024 | .981 | 0.000 |
|  |  |  |  |  |  |
| Model 2: Language Comprehension (*a*^2^ path) | | |  |  |  |
| 1. Constant | -1.640 (0.834) | — | -1.967 | .052 | — |
| 2. Audiovisual Integration | 3.120 (0.584) | .544 | 6.203 | <.001*** | 1.302 |
| 3. Age | -0.002 (0.005) | .018 | -0.388 | .699 | 0.000 |
|  |  |  |  |  |  |
| Model 3: Reading Comprehension (*b* and *c’* paths) | | | | | |
| 1. Constant | -0.159 (0.236) | — | -0.067 | .583 | — |
| 2. Audiovisual Integration | -0.013(0.165) | -.005 | -0.079 | .937 | 0.000 |
| 3. Decoding | 0.314 (0.086) | .297 | 3.662 | <.001*** | 0.097 |
| 4. Language Comprehension | 0.282 (0.040) | .613 | 7.054 | <.001*** | 0.602 |
| 5. Age | 0.001(0.002) | .039 | 0.736 | .464 | 0.002 |
|  |  |  |  |  |  |
| Completely standardized indirect effect through decoding 95% CI: [0.057, 0.223] | | | | | |
| Completely standardized indirect effect through language comprehension 95% CI: [0.207, 0.471] | | | | | |
| Completely standardized parallel indirect effect 95% CI: [0.309, 0.605] | | | | | |

*Note*. Coefficients, *p* values, and *f ^2^* values for regression analyses. *f* ^2^ ≥ .02 indicates a small effect size, *f* ^2^ ≥ .15 indicates a moderate effect size, *f* ^2^ ≥ .35 indicates a large effect size (Cohen, 1988).

**p* value for effect < .05, ***p* value for effect < .01, ****p* value for effect < .001.

**Table S3**

*Summary of Parallel Mediation Model with NVIQ Included as a Covariate*

| Model/Variable | *B* (SE) | *β* | *t* | *p* | *f* ^2^ |
| --- | --- | --- | --- | --- | --- |
| Model 1: Decoding (*a*^1^ path) | | |  |  |  |
| 1. Constant | -2.808 (0.584) | — | -4.808 | <.001*** | — |
| 1. Audiovisual Integration | 0.728 (0.234) | .291 | 3.117 | .002** | 0.097 |
| 1. NVIQ | 0.021 (0.005) | .353 | 3.783 | <.001*** | 0.142 |
|  |  |  |  |  |  |
| Model 2: Language Comprehension (*a*^2^ path) | | |  |  |  |
| 1. Constant | -6.099 (1.270) | — | -4.801 | <.001*** | — |
| 1. Audiovisual Integration | 2.394 (0.508) | .319 | 4.710 | <.001*** | 0.113 |
| 1. NVIQ | 0.041 (0.012) | .351 | 3.401 | .001** | 0.141 |
|  |  |  |  |  |  |
| Model 3: Reading Comprehension (*b* and *c’* paths) | | | | | |
| 1. Constant | -0.490 (.959) | — | -1.167 | .246 | — |
| 1. Audiovisual Integration | -0.029(.164) | -.011 | -0.176 | .861 | 0.000 |
| 1. Decoding | 0.297 (.087) | .281 | 3.418 | <.001*** | 0.086 |
| 1. Language Comprehension | 0.275 (.040) | .598 | 6.883 | <.001*** | 0.557 |
| 1. NVIQ | 0.005(.004) | -.072 | 1.197 | .235 | 0.000 |
|  |  |  |  |  |  |
| Completely standardized indirect effect through decoding 95% CI: [0.023, 0.161] | | | | | |
| Completely standardized indirect effect through language comprehension 95% CI: [0.135, 0.381] | | | | | |
| Completely standardized parallel indirect effect 95% CI: [0.183, 0.475] | | | | | |

*Note*. Coefficients, *p* values, and *f ^2^* values for regression analyses. *f* ^2^ ≥ .02 indicates a small effect size, *f* ^2^ ≥ .15 indicates a moderate effect size, *f* ^2^ ≥ .35 indicates a large effect size (Cohen, 1988).

**p* value for effect < .05, ***p* value for effect < .01, ****p* value for effect < .001.

**Table S4**

*Summary of Relations Between All Indices Derived from the Psychophysical Task and Outcomes of Interest According to Group*

|  | Autism | Non-Autism | Total |
| --- | --- | --- | --- |
| Magnitude of Associations with Visual-Only Accuracy | | | |
| 1. Decoding | -.007 | .239 | .079 |
| 1. Language Comprehension | -.129 | .083 | -.027 |
| 1. Reading Comprehension | -.084 | .188 | .030 |
|  |  |  |  |
| Magnitude of Associations with Auditory-Only Accuracy | | | |
| 1. Decoding | -.049 | -.031 | -.035 |
| 1. Language Comprehension | .069 | -.012 | .024 |
| 1. Reading Comprehension | .014 | .019 | .014 |
|  |  |  |  |
| Magnitude of Associations with Congruent Audiovisual Accuracy | | | |
| 1. Decoding | .063 | .234 | .112 |
| 1. Language Comprehension | .078 | .057 | .047 |
| 1. Reading Comprehension | .116 | .024 | .065 |
|  |  |  |  |
| Magnitude of Associations with Audiovisual Integration | | | |
| 1. Decoding | .147 | .439 | .430 |
| 1. Language Comprehension | .285 | .279 | .535 |
| 1. Reading Comprehension | .329 | .151 | .459 |

*Note*. Values summarized in the table represent standardized coefficients from regression analyses.
